# Supplementary material for: Splice-Junction-Based Mapping of Alternative Isoforms in the Human Proteome
Source: Cell Rep. Author manuscript; Available in PMC 2020 Jan 15. (PMC6961840; doi:10.1016/j.celrep.2019.11.026)
Supplement: 2 [file NIHMS1546469-supplement-2.zip › DF1/PXD006675/RightVentricle/RightVentricle_36_NEXN_QEMGEEDDVDVRPAR.pdf]

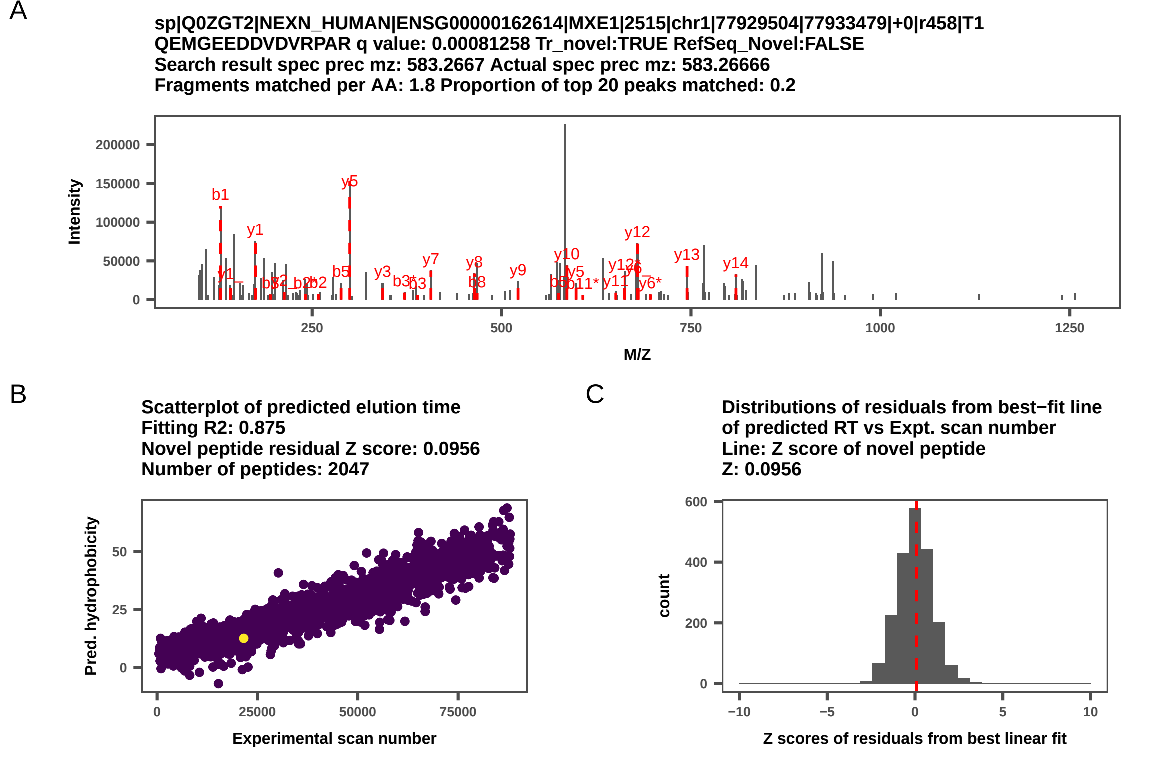
A
sp|Q0ZGT2|NEXN_HUMAN|ENSG00000162614|MXE1|2515|chr1|77929504|77933479|+0|r458|T1
QEMGEEDDVDVRPAR q value: 0.00081258 Tr_novel:TRUE RefSeq_Novel:FALSE
Search result spec prec mz: 583.2667 Actual spec prec mz: 583.26666
Fragments matched per AA: 1.8 Proportion of top 20 peaks matched: 0.2
Intensity
200000
150000
100000
50000
0
b1
y1
y5
b5
y3
y7
y8
b8
y9
y10
y12
y13
y14
250
500
750
1000
1250
M/Z
B
Scatterplot of predicted elution time
Fitting R2: 0.875
Novel peptide residual Z score: 0.0956
Number of peptides: 2047
Pred. hydrophobicity
50
25
0
0
25000
50000
75000
Experimental scan number
C
Distributions of residuals from best−fit line
of predicted RT vs Expt. scan number
Line: Z score of novel peptide
Z: 0.0956
count
600
400
200
0
−10
−5
0
5
10
Z scores of residuals from best linear fit
